# Solving Minimum Spanning Tree Problems Based on DNA Chemical Reaction Networks

**DOI:** 10.2174/0113862073360358250128094601

**Published:** 2025-03-18

**Authors:** Jing Yang, Yawen Zheng, Zhixiang Yin, Xianya Geng, Zhen Tang

**Affiliations:** 1 School of Mathematics and Big Data, Anhui University of Science and Technology, Huainan, China;; 2 School of Mathematics, Physics and Statistics, Shanghai University of Engineering Science, Shanghai, China

**Keywords:** DNA chemical reaction network, minimum spanning tree, prim algorithm, DNA strand displacement, threshold module, molecular dynamics

## Abstract

**Introduction:**

DNA strand displacement reactions are emerging as a promising biocomputing tool. The minimum spanning tree problem is fundamental in graph theory. This paper explores the use of DNA strand displacement reaction networks for addressing the minimum spanning tree problem. We also present a computing model that is based on DNA strand displacement reactions.

**Methods:**

The model effectively solves the minimum spanning tree problem by intelligently integrating the three reaction modules of weighted, threshold, and sum. Thus, initially, we encoded the edges in the graph using distinct DNA sequences and effectively assigned the edges their respective weights. Afterwards, the threshold module applied a filter to the weighted edges based on the fluorescence intensity. Ultimately, the sum module gathered the filtered edges to calculate the overall weight of the minimum spanning tree. In order to verify the effectiveness of the proposed method, we conducted simulation experiments using visual DSD software.

**Results:**

The results of the simulations showed the viability and precision of this DNA computing model in resolving intricate problems.

**Conclusion:**

Furthermore, this study not only confirms the capability of DNA computing in solving problems related to graph theory, but also offers significant theoretical backing and experimental foundation for the future advancement of DNA-based computer systems and biocomputing applications.

## 1 INTRODUCTION

As time progresses, scientific and technological advancement occurs, and a variety of intricate computational problems, particularly those of the NP type, continue to arise. These issues present a significant challenge to the capabilities of modern computers. Consequently, the current research is mostly focused on the advancement of high-performance computers. In 1994, Adleman's study [1] introduced DNA computers to the world. Subsequently, the field of DNA computers has garnered the interest of numerous researchers and swiftly entered a phase of robust advancement. In 1995, Lipton [2] expanded the computational approach to encompass the determination of the satisfiability of Boolean formulas, so ushering in a novel era of DNA molecular-based circuits. In 2004, Okamoto *et al.* [3] successfully integrated digital circuits with DNA computing. This breakthrough allowed for the development of general-purpose DNA computing, expanding the scope of research in this field. In recent years, researchers have been actively investigating the combination of DNA computing with intelligent computing techniques, such as genetic algorithms [4-6], neural networks [7-9], fuzzy systems [10-13], and chaotic systems [14-18]. This emerging trend underscored the distinct benefits of DNA computing.

Research on DNA computing has explored several elements, like as computational capabilities, computational models, and algorithm design, in recent years. DNA Strand Displacement (DSD) reactions are a significant way of DNA computing that can enable the creation of sophisticated chemical reaction networks [19]. This is due to their ability to store large amounts of information [20], operate in parallel [21, 22], and be easily accessed. The combination of DNA strand displacement technology with self-assembly and fluorescent signal labelling has led to the development of a range of applications, including DNA logic gates [10, 23-33], sensors [34-39], molecular detection [40-45], DNA nanorobotics [46-50], DNA nanostructures [51-59], smart drug delivery [60-64], disease diagnosis, and therapy [65-72]. In 2018, Cherry [73] and her colleagues developed a winner-take-all neural network by using DNA strand displacement processes. In 2020, a DNA strand displacement-based circuit was used to perform digital operations by Wang *et al*. [74]. In contrast to conventional logic gate circuits, the DNA-based switching circuit had already exhibited a more efficient and simplified structure by that time. In 2021, Emanuelson *et al.* [75] combined DNA computing with optical control to create a groundbreaking NOT logic gate that operates based on optical responses. This method enhanced both the responsiveness of DNA logic gates and the flexibility and manipulability of control. In 2022, Lapteva *et al.* [76] presented a method for storing information in DNA-based devices throughout time. In 2024, Huang *et al.* [77] developed a method for constructing logic circuits for DNA strand displacement. This strategy aimed to enhance the flexibility and modularity of the circuits, enabling them to perform sophisticated logic functions. This interdisciplinary fusion not only brought new impetus to the field of intelligent computing, but also expected to provide more efficient solutions for complex problems through this research.

As the network grows larger and more complicated, the minimum spanning tree problem, which is a fundamental problem in graph theory, will encounter more significant obstacles and possibilities. Hence, additional, comprehensive investigation into the solution techniques for minimum spanning trees is necessary to discover more effective and reliable algorithms to address the requirements in real-world scenarios. In 2008, a method for representing and solving the minimum spanning tree problem using DNA computation was proposed in the literature [78]. The approach integrates the benefits of DNA computing and genetic algorithms, utilizing a set length to efficiently encode and decode the solution of the minimum spanning tree. In 2012, Zhou *et al.* [79] sought to overcome the limitations of traditional computational methods for specific problems. They accomplished this by integrating biochemical experiments with the minimum spanning tree problem in graph theory. Their aim was to leverage the significant parallelism inherent in biochemical experiments to enhance computational efficiency. In 2023, Zhong *et al.* [80] released an article that showcased the efficacy and feasibility of the minimum spanning tree algorithm in addressing real-world issues.

The article presents a chemical reaction network model using DNA strand displacement. This model offers a fresh approach to addressing the classical minimum spanning tree problem in computer science. Conventional computer methods are effective but do not possess the intuitive and adaptable nature of biomimicry for addressing these issues. The DNA strand displacement methodology is a novel molecular computational tool that offers potential solutions to intricate challenges due to its distinctive biocompatibility and dynamic tunability. This study presents the Prim algorithm, a well-known graph-theoretic procedure, as a method to connect these DNA strand displacement response modules and solve the minimum spanning tree problem. To verify the design of this work, simulations are conducted using the Visual DSD program.

## 2 DNA CHEMICAL REACTION NETWORKS AND THE MINIMUM SPANNING TREE PROBLEM

### 2.1 DNA Strand Displacement

The DNA Strand Displacement (DSD) reaction is a crucial component of the DNA chemical reaction network. The DNA strand displacement reaction is a unique reaction that operates on the principles of free energy difference and complementary pairing in DNA molecular hybridization. During a DNA strand displacement process, when a single-stranded DNA molecule establishes a strong hydrogen connection with one of the two strands in a double-stranded DNA molecule, it has the ability to replace or push out the other strand of the double-stranded DNA molecule. During this process, the single-stranded DNA serves as an "intruder," progressively displacing the original DNA strand and thereby altering the structure of the DNA molecule. The fundamental concept of the reaction is illustrated in Fig. (**1**). The DNA strand displacement reaction offers several benefits, including high selectivity, high sensitivity, high efficiency, and low cost. Consequently, it has found extensive application in the domains of molecular biology and bioanalysis.

### 2.2 Minimum Spanning Tree Problem

The Minimum Spanning Tree (MST) problem involves identifying a tree that includes all vertices in a weighted undirected graph, while minimizing the total weight of all edges in the tree. For instance, in an undirected graph *G*=(*V,E*), the edge *e*=(*u,v*) denotes the connection between vertex *u* and vertex *v*, while *w*(*u,v*) reflects the weight of this edge. If there exists a subset *T* of the graph *G* that forms a no-loop graph, and the weight of the minimal spanning tree *w*(*T*) within *T* connects all the vertices of *G*, then *T* is the minimum spanning tree.

The minimum spanning tree is widely used in network architecture, communication line planning, data compression, and other domains. The minimum spanning tree not only signifies the most economical approach to building a network, but also symbolizes the notion of allocating resources optimally. Hence, the examination of the minimum spanning tree possesses not only crucial theoretical significance but also holds a prominent position in practical implementations. Due to the ongoing advancements in computer technology, the solution approach for finding the minimum spanning tree has undergone continual optimization and enhancement. The Kruskal algorithm, Borůvka algorithm, and Sollin algorithm have distinct advantages in various application contexts, starting from the initial Prim algorithm. However, as the size and complexity of networks continue to escalate, finding a swift and precise solution for the minimum spanning tree has emerged as a prominent area of focus in contemporary research.

### 2.3 Prim Algorithm

The prim algorithm is a graph theory algorithm that seeks to find minimum spanning trees in weighted linked graphs. This algorithm can commence from any vertex in the graph. At each iteration, it chooses the edge with the lowest weight that connects to all the vertices in the current set of vertices. It then includes the vertex from that edge into the current set of vertices. This process is repeated iteratively until all vertices have been incorporated, resulting in the formation of the minimum spanning tree. The process is as follows:

1. Input: Given a weighted connected graph with a vertex set *V* and an edge set *E*;
2. Initialisation: Initialize *V_new_* as a set containing a single vertex, *x* (the starting point), from *V*, and *E_new_* as an empty set;
3. Continuously perform the following operations until the value of *V_new_* is equal to *V*:
  - Choose the edge 〈*u,v*〉 from the set *E* that has the smallest weight. Here, *u* is an element in the set *V_new_* and *v* is not in the set *V_new_*, but *v* is an element of *V*. If there are multiple edges that satisfy this condition and have the same weight, any one of them can be chosen arbitrarily.
  - Add *v* to the set *V_new_* and add the 〈*u,v*〉 edge to the set *E_new_*;

4) Output: Describe the resulting minimum spanning tree using the sets *V_new_* and *E_new_*.

## 3 MATERIALS AND METHODS

### 3.1 Model Building

When constructing a graph model, it is customary to assign a weight to each edge. This weight represents a specific metric or degree of relationship between two vertices of a connection. These weights are crucial in graph-theoretic methods that solve the minimum spanning tree problem as they directly influence the priority or cost of constructing the tree.

The Prim algorithm is a highly efficient approach for tackling the minimum spanning tree problem. We can broadly categorize the algorithm into the following three steps. Firstly, we perform a weighted assignment, where each edge in the graph is given a corresponding weight. Next, we select a vertex as the starting point and, using a threshold-based selection process, choose the edge with the lowest weight among all edges currently connected to the set of selected vertices. This edge is then included in the spanning tree. Subsequently, we repeat this selection process, incorporating additional vertices and their corresponding lowest-weight edges into the spanning tree, until all vertices are included within the spanning tree. Finally, we obtain the solution of the minimum spanning tree by calculating the sum of the weights of all selected edges. The comprehensive solution methodology is illustrated in Fig. (**2**).

#### 3.1.1 Weighted Reaction Module

Our primary focus in designing this module is to execute a weighting operation on every edge within the graph. Upon entry into the weighted reaction module, single-stranded DNA (edge AB) with a concentration of 1 nm is introduced, engaging in a DSD reaction with double-stranded DNA (S). This reaction yields single-stranded DNA (W) and double-stranded DNA (waste1), under the assumption that the single-stranded DNA represents the edges within the graph. Subsequently, the single-stranded DNA (W) reacts with the composite DNA strand (N), leading to the production of single-stranded DNA (edge AB) with a concentration of K nm. This process enables us to assign precise weight values to each edge. It is crucial to note that when an edge's weight is 1, it signifies a uniform weight in the construction of the minimum spanning tree. In this case, it does not require processing through the weighted reaction module, and can be directly passed to the threshold reaction module for further operations. The reaction process is shown in Fig. (**3**), and the ideal chemical reaction formula is:


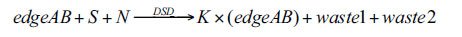


#### 3.1.2 Threshold Reaction Module

The threshold reaction module is a crucial component of our overall model, tasked primarily with filtering out the edges with the smallest weights from the output of the weighted reaction module. Specifically, we input the K nm single-stranded DNA (edge AB) generated by the weighted reaction module into the threshold reaction module, where it undergoes a DSD reaction with double-stranded DNA (G), yielding fluorescently labeled single-stranded DNA (edge YAB). We detect the intensity of the fluorescent signal using fluorescence detection equipment (fluorescence spectrophotometer or fluorescence microscope). The intensity of the fluorescence signal is proportional to the concentration of the target DNA strand, and the intensity of the fluorescence signal is used to distinguish between strands of different weights. This methodology enables us to pinpoint the edge or edges with the lowest fluorescence levels, which are fundamental for constructing the minimum spanning tree. In scenarios where the threshold reaction module identifies multiple edges sharing the same, current minimum weight, we simultaneously consider all such edges as candidate edges. This approach underscores the inherent parallelism advantage of DNA computing, empowering our system to process multiple pathways concurrently, thereby augmenting screening efficiency.

In the first stage, the weighted reaction module performs the dual role of selecting edges and assigning them weights. Subsequently, the threshold reaction module takes over, selecting the edges with the lowest weights from the pool of weighted edges, and utilizing them as the foundational inputs for constructing the minimum spanning tree. The reaction process is shown in Fig. (**4**), and the ideal chemical reaction formula is:


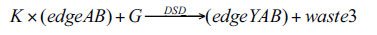


#### 3.1.3 Sum Reaction Module

We utilize the threshold reaction module to select the edges with the lowest weights from among all the edges that connect to the currently unselected vertices. Following this selection, the sum reaction module performs a summing operation on the chosen edges, calculating their cumulative weights. For the purpose of the DSD reaction involving double-stranded DNA (P), we direct all the screened single-stranded DNAs into the Sum reaction module. This reaction process generates both single-stranded DNA (O) and double-stranded DNA (waste4). Ultimately, the concentration of single-stranded DNA (O) corresponds precisely to the total weight required for constructing the minimum spanning tree. The reaction process is shown in Fig. (**5**) and the ideal chemical reaction formula is:


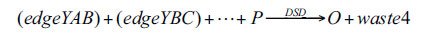


### 3.2 Example Analysis

To ensure simplicity, we validate the model by examining a network comprising of 5 nodes, as depicted in Fig. (**6**). We present the weighted graph in Table **1**.

According to the Prim algorithm, we can choose any vertex in the network as the initial point. If vertex *A* is chosen as the initial point, we must take into account all the edges linked to it. Subsequently, the weighted reaction module assigns weights to all the edges that are related to *A*. The edges connecting to vertex *A* are *A-B* and *A-C*. To conduct simulation experiments, we utilize Visual DSD. Fig. (**7**) displays the results of our simulations.

We use the weighted reaction module to assign weights to all the edges connected to *A*. It is evident that the edges *A-B* and *A-C* are successfully assigned weights of 2 nm and 3 nm, respectively. Furthermore, we require the threshold reaction module to further determine the edge that must be included in the minimum spanning tree. Fig. (**8**) displays the outcomes of our simulation. It is evident that the threshold reaction module was successful in converting the edges *A-B* and *A-C* into equal concentrations of fluorescently labelled DNA strands YAB (sp11) and YAC (sp7). Our findings indicate that the weight of the *A-B* edge is 2, which is the lowest among all edges connected to *A*. Thus, based on the Prim algorithm, we chose the *A-B* edge as the initial extended edge for the minimum spanning tree.

Next, we need to repeat the weighted reaction module with the threshold reaction module until all vertices are interconnected. Fig. (**9**) depicts the entire process of these simulations. Specifically, for the vertices *A* and *B* in the *set{A,B}*, we must identify the edges YAC(sp28)、YBE(sp30)、YBD(sp21) and YBC(sp17) Among these, we select the edge with the lowest weight, which is *B-C* with a weight of 1. Following this, we add *C* to the set of the minimum spanning tree. Then, we proceed to identify the edges YBE(sp30)、YBD(sp21)、YCE(sp23) and YCD(sp14) that connect to the vertices in the *set{A,B,C}*. After evaluating these edges through weighted and threshold considerations, we determine that *B-E* has the lowest weight (weight 3). We then connect edges *B* and *E*, adding *E* to the list of minimum spanning trees. Subsequently, we look for the lowest-weight edge that connects to *D* from the {*A,B,C,E*}. Among YBD(sp9) and YCD(sp5), we find that *B-D* has a weight of 5, which is the lowest. We connect the edges *B-D* and include *D* in the list of minimum spanning trees. At this point, all vertices have been successfully incorporated into the minimum spanning tree.

Finally, we obtain the conclusive solution from the sum reaction module. The total weight of the minimum spanning tree for this graph is 11(sp12). We display the outcomes of the simulation in Figs. (**10** and **11**).

The above example demonstrates that we can successfully determine the minimum spanning tree of a weighted graph.

Although a total of 32 different DNA strands are used in solving a 5-node weighted graph, the parallelisability, spontaneity, and scalability of the DNA strand displacement technique means that when faced with more complex graphs, its solution efficiency does not increase in a linear fashion with the number of DNA strands. In the traditional computational methods employed for the resolution of the minimum spanning tree problem, the time complexity is typically n^2^, which may result in inefficiency when confronted with large-scale datasets. Furthermore, these methods necessitate additional storage space to maintain the adjacency matrix and the structure of the minimum spanning tree. In contrast, computational techniques based on DNA strand substitution exhibit notable programmability, enabling the attainment of diverse computational paths through the adjustment of the DNA sequence. Concurrently, the information storage capacity of the technique allows for its adaptation to varying computational requirements, thus achieving substantial efficiency gains.

## 4 RESULTS

However, as the number of vertices in the graph grows, not only does the configuration of the minimum spanning tree necessitate reassessment, but also the implications of this change on computational complexity and experimental execution must be contemplated. The structure of the minimum spanning tree becomes more susceptible to modification as the number of vertices in the weighted undirected graph increases. To maintain connectivity, the newly introduced vertices must be interconnected with others, which might prompt the selection of novel edges or adjustments to the initial optimal edge choice. We can see that the experiment reaches equilibrium in about 5000s from the simulation experiments we have carried out. Moreover, when the vertex count surges, the minimum spanning tree algorithm is compelled to traverse all edges and make decisions based on their weights, thereby intensifying the edge count, expanding the data volume for processing, and potentially prolonging the calculation duration.

Nevertheless, the parallel processing of a large number of DNA strands can considerably accelerate the computational speed. However, this parallelism also introduces a heightened level of complexity to the system, necessitating meticulous control and synchronisation of a multitude of concurrent reactions. Additionally, if a significant portion of the graph's edges share the same weight, additional data structures might be indispensable to maintain the edge organization, further demonstrating the advantages of the model when dealing with large-scale data.

## 5 DISCUSSION

The chemical reaction network based on DNA strand displacement proposed in this study provides a new perspective to solve the classical minimum spanning tree problem in computer science. In the study, we utilize a distinct DNA sequence to encode each node and edge. We establish the connection between edges in the graph using a weighted reaction network. We then employ fluorescence technology to pick weighted edges. Finally, we determine the overall value by summing the response module. Using Visual DSD software, we effectively solve a minimum spanning tree problem with 5 nodes by modeling the process of DNA strand displacement reaction and tracking the change in DNA strand concentration. This result not only confirms the practicality of the computational approach of DNA strand displacement, but also offers robust technological assistance for us to delve deeper into larger size or more intricate minimum spanning tree problems.

## CONCLUSION

Nevertheless, we acknowledge that the practical implementation of the DNA strand displacement technique is currently confronted with numerous obstacles. For instance, the occurrence of a leakage response can disrupt the calculating procedure and compromise the precision of the outcomes. Furthermore, the intricacy of DNA strand design, the intricacy of molecular dynamics, and the intricate interactions during the reaction process can result in discrepancies in the calculation findings. Hence, in future research, it is imperative to enhance the DNA strand design and enhance the precision of the reaction process to attain accurate design and management of extensive, complicated reaction networks.

Notwithstanding these challenges, the capacity of DNA strand displacement technology to address intricate computational problems must not be disregarded. In contrast to conventional computational methods, it exhibits greater adaptability and expandability to manage extensive datasets within particular circumstances. Hence, we posit that as technology advances and research deepens, DNA strand displacement technology will assume a progressively vital role in molecular computing, nanotechnology, biocomputing, and related domains, thereby paving the way for future scientific inquiry and technological breakthroughs.

## Figures and Tables

**Fig. (1):**
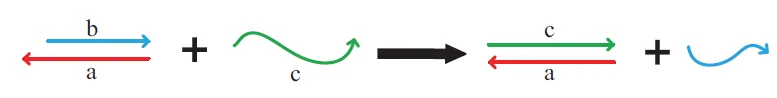
Basic reaction principle of DSD.

**Fig. (2):**
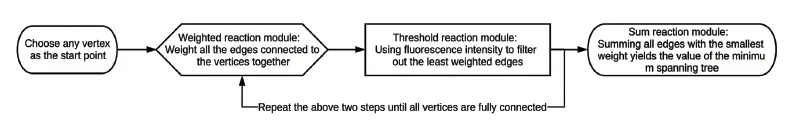
Flowchart for solving the minimum spanning tree.

**Fig. (3):**
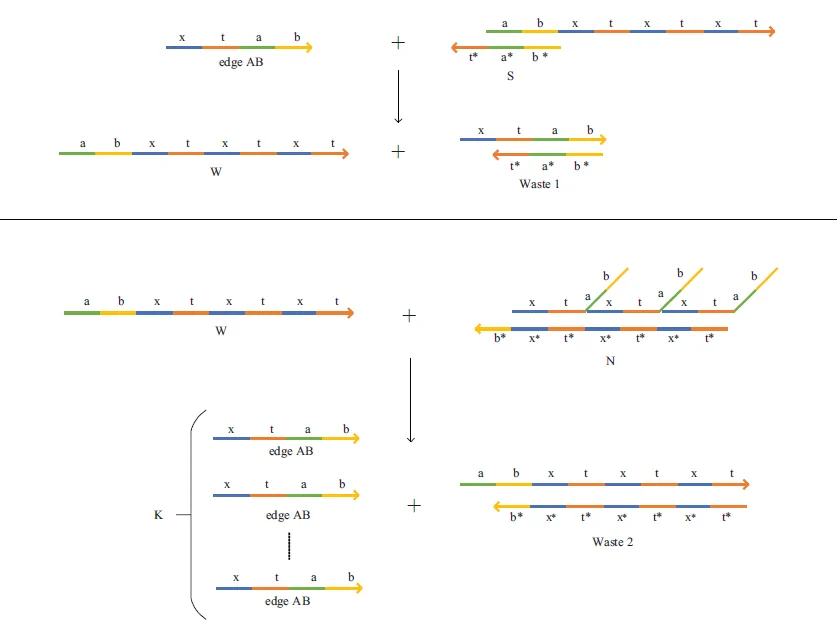
Weighted reaction module.

**Fig. (4):**
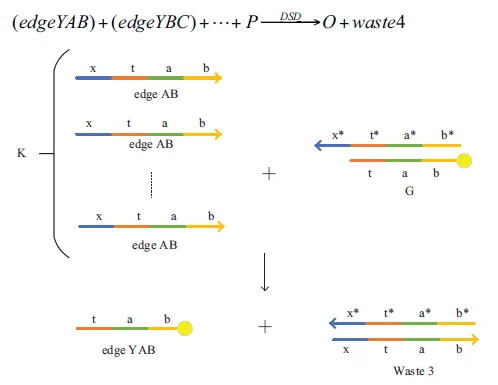
Threshold reaction module.

**Fig. (5):**
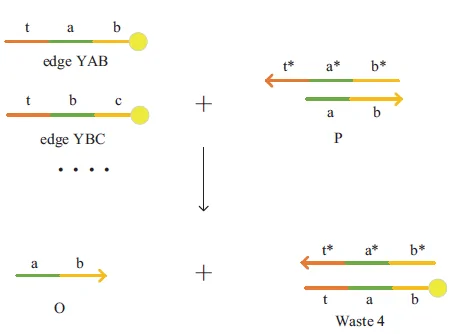
Sum reaction module.

**Fig. (6):**
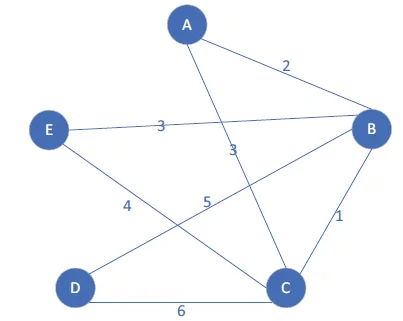
5-node weighted graph.

**Fig. (7):**
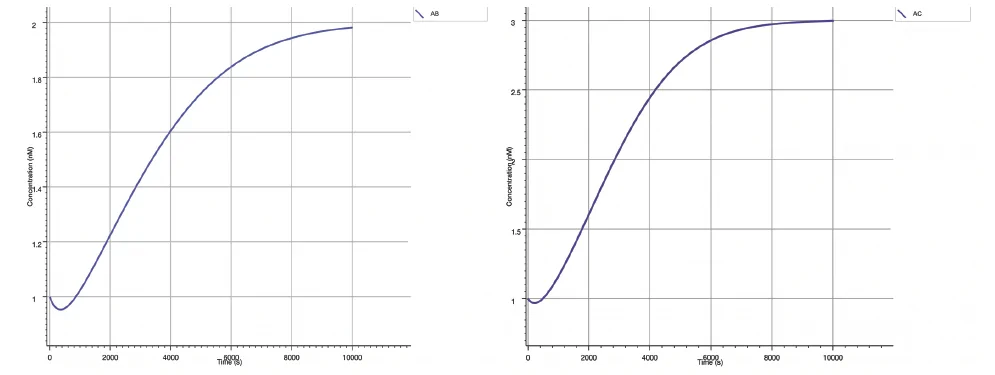
The complete simulation time series graph of the edges A-B and A-C weighted reaction module.

**Fig. (8):**
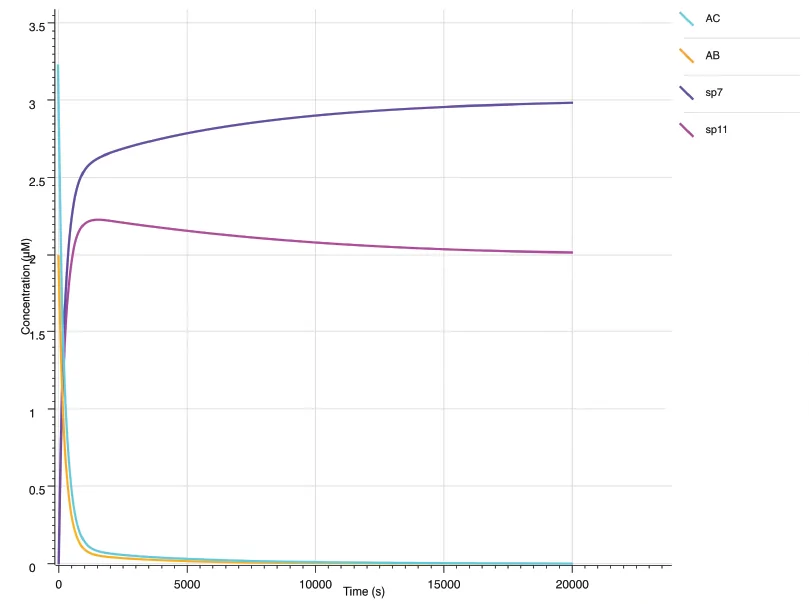
The complete simulation time series graph of the edges A-B and A-C threshold reaction module.

**Fig. (9):**
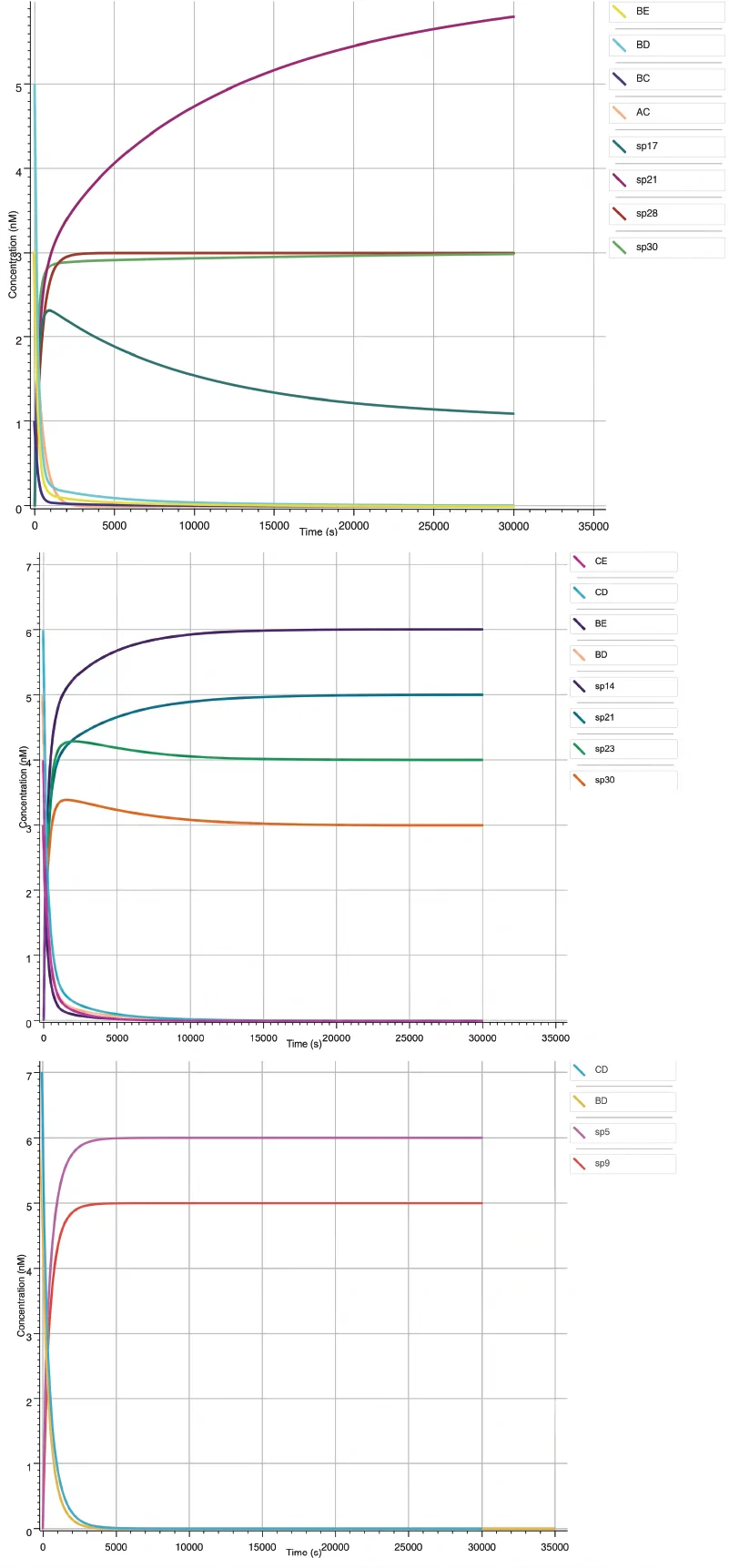
The complete simulation time series graph of the remaining edges threshold reaction module.

**Fig. (10):**
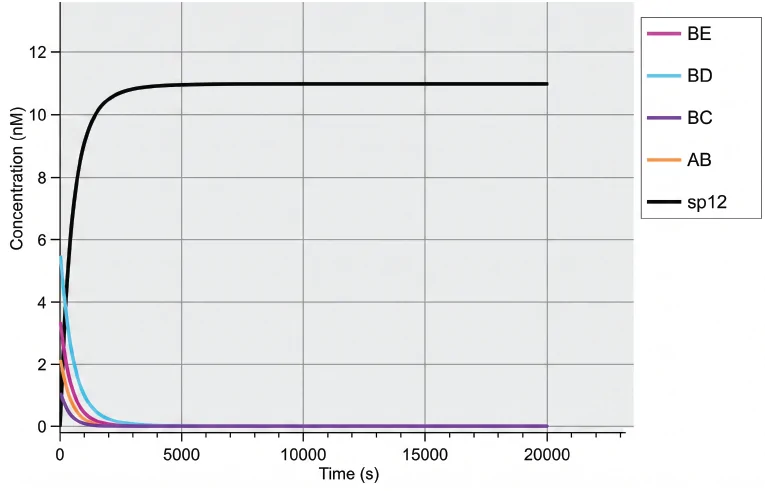
The concentration curve of the sum reaction module.

**Fig. (11):**
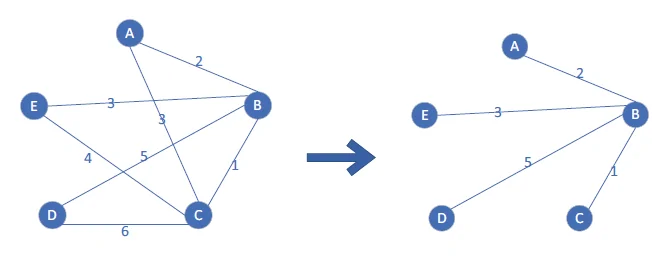
5-node minimum spanning tree.

**Table 1:** 5-node weighted graph.

**S. No.**	**Edge**	**Weight**
1.	A-B	2
2.	A-C	3
3.	B-C	1
4.	B-D	5
5.	B-E	3
6.	C-E	4
7.	C-D	6

## Data Availability

The original contributions presented in the study are included in the article. Further inquiries can be directed to the corresponding author.

## LIST OF ABBREVIATIONS

DSD: DNA Strand Displacement
MST: Minimum Spanning Tree
